# Optimizing acupuncture treatment for dry eye syndrome: a systematic review

**DOI:** 10.1186/s12906-018-2202-0

**Published:** 2018-05-03

**Authors:** Bong Hyun Kim, Min Hee Kim, Se Hyun Kang, Hae Jeong Nam

**Affiliations:** 1 Department of Ophthalmology and Otolaryngology of Korean Medicine, College of Korean Medicine, Kyung Hee University, 26, Kyungheedae-ro, Dongdaemun-gu, Seoul, 02453 Republic of Korea; 2 Department of Ophthalmology and Otolaryngology of Korean Medicine, Kyung Hee University Korean Medicine Hospital, 23, Kyungheedae-ro, Dongdaemun-gu, Seoul, 02447 Republic of Korea; 3 Department of Ophthalmology and Otolaryngology of Korean Medicine, Kyung Hee University Hospital at Gangdong, 892, Dongnam-ro, Gangdong-gu, Seoul, Republic of Korea

**Keywords:** Dry eye syndrome, Acupuncture, Systematic review

## Abstract

**Background:**

In a former meta-analysis review, acupuncture was considered a potentially effective treatment for dry eye syndrome (DES), but there were heterogeneities among the outcomes. We updated the meta-analysis and conducted subgroup analysis to reduce the heterogeneity and suggest the most effective acupuncture method based on clinical trials.

**Methods:**

We searched for randomized controlled trials (RCTs) in 10 databases (MEDLINE, EMBASE, CENTAL, AMED, SCOPUS, CNKI, Wangfang database, Oriental Medicine Advanced Searching Integrated System (OASIS), Koreamed, J-stage) and searched by hand to compare the effects of acupuncture and artificial tears (AT). We also conducted subgroup analysis by (1) method of intervention (acupuncture only or acupuncture plus AT), (2) intervention frequency (less than 3 times a week or more than 3 times a week), (3) period of treatment (less than 4 weeks or more than 4 weeks), and (4) acupoints (BL1, BL2, ST1, ST2, TE23, Ex-HN5). The Bucher method was used for subgroup comparisons.

**Results:**

Nineteen studies with 1126 patients were included. Significant improvements on the Schirmer test (weighted mean difference[WMD], 2.14; 95% confidence interval[CI], 0.93 to 3.34; *p* = 0.0005) and break up time (BUT) (WMD, 0.98; 95% CI, 0.79 to 1.18; *p* < 0.00001) were reported. In the subgroup analysis, acupuncture plus AT treatment had a weaker effect in BUT but a stronger effect on the Schirmer test and a better overall effect than acupuncture alone. For treatment duration, treatment longer than 1 month was more effective than shorter treatment. With regard to treatment frequency, treatment less than three times a week was more effective than more frequent treatment. In the acupoint analysis, acupuncture treatment including the BL2 and ST1 acupoints was less effective than treatment that did not include them. None of those factors reduced the heterogeneity.

**Conclusions:**

Acupuncture was more effective than AT in treating DES but showed high heterogeneity. Intervention differences did not influence the heterogeneity.

## Background

Dry eye syndrome (DES) is a multifactorial disease of the tears and ocular surface that can result in ocular discomfort and visual impairment [1]. In recent years, the geriatric proportion of the overall population has increased, and factors causing eye fatigue have diversified (e.g., excessive use of computers or smartphones). In 2011, the prevalence of DES in South Korea was 8.0% [2]. According to statistics from the National Health Insurance Service in South Korea, the prevalence of DES is increasing continuously “(http://opendata.hira.or.kr/home.do)”.

Acupuncture can alleviate DES. Some systematic reviews showed that acupuncture is effective for DES [3–5]. However, heterogeneous results from various interventions made it difficult to draw clear conclusions. Each study used different acupoints, durations, and frequencies. An individual treatment strategy according to patient condition is ideal. However, an individual treatment strategy depends on each clinician’s subjective experience, which indicates low effectiveness of novice practitioners. An adequate acupuncture dose is needed to standardize and optimize its effects. Also, some standards are required to increase the reproducibility of results. There have been some attempts to define adequate acupuncture treatments for other diseases [6, 7].

Our aim in this study was to evaluate the efficacy of acupuncture using the latest research and to suggest a standard acupuncture treatment for patients with DES, including acupoints, number of sessions, and treatment duration. Therefore, we conducted a systematic review of published acupuncture treatments for DES and analyzed the factors that influence therapeutic effectiveness.

## Methods

### Search strategy

We searched MEDLINE, EMBASE, CENTAL, AMED, SCOPUS, CNKI, the Wangfang database, the Oriental Medicine Advanced Searching Integrated System (OASIS), Koreamed, and J-stage and conducted manual searches for potentially relevant articles published through July 2017. (CNKI and Wangfang are Chinese databases. OASIS and Koreamed are Korean databases. J-stage is a Japanese database.) The search terms used were ‘acupuncture’ AND (‘dry eye’ OR ‘xerophthalmia’ OR ‘keratoconjunctivitis sicca’). In the Chinese databases (CNKI, Wangfang database), we used the Chinese terms (‘干眼’ AND ‘针’). There were no limits with regard to publications, other than language limits of English, Chinese, or Korean.

### Study selection

The specific inclusion criteria were as follows: (1) clinical trial for DES patients; (2) use of acupuncture or an applied form (e.g., electroacupuncture, pyonex); (3) control group that received appropriate placebo or artificial tears (AT); (4) outcomes included Schirmer’s test (ST), break up time test (BUT), or corneal fluorescein staining (CFS); (5) randomized controlled trials (RCT); and (6) full text available. We excluded (1) studies that included Sjögren syndrome patients and (2) interventions combined with other treatments (e.g., herbal medicine, moxibustion). Two researchers (MHK and SHK) carried out the study selection independently and discussed their differences.

### Data extraction and assessment of risk of bias

Three independent reviewers (MHK, BHK and SHK) read all selected articles. We extracted publication data, participant information, intervention regime (sites, duration, and frequency), outcome measures, and drop-outs. After extraction, we assessed the risk of bias using the Cochrane Collaboration tool [8].

### Data analysis

For our meta-analysis of similar treatment interventions, we used the statistical software provided by the Cochrane Collaboration (RevMan 5.3). The estimated effect of the data was calculated using the weighted mean difference (WMD) and confidence interval (CI). The Q-test or χ^2^ was used to evaluate heterogeneity [9]. When the compared populations were homogeneous (Q-test *p* > 0.1), we used the fixed-effect model; when they were heterogeneous (*p* < 0.1), we used the random-effect model. We also performed a sensitivity analysis. Each study was sequentially excluded from the meta-analysis, and the sensitivity was determined from the corresponding heterogeneity results. Funnel plot was conducted for detecting publication bias.

In our subgroup analysis, we used the standard mean difference (SMD) in BUT and ST values to evaluate the overall effects according to the Bucher method. This is one of the most suitable indirect comparisons for RCTs. We applied this method, as there were no direct comparative trials and an indirect method can provide useful information for optimization. This method is supposed that treatments A and C are compared in one RCT and treatments B and C are compared in another RCT, the indirect comparison of A and B is adjusted according to C (common comparator). This method assumes that indirect evidence is consistent with a direct comparison [10].

We analyzed the interventions as follows: (1) method of intervention (acupuncture only or acupuncture plus AT), (2) intervention frequency (less than 3 times a week or more than 3 times a week), (3) treatment duration (less than 4 weeks or more than 4 weeks), and (4) acupoints (BL1, BL2, ST1, ST2, TE23, Ex-HN5). Selection of the acupoints frequently used in clinical trials is explained in a previous study [11].

## Results

### Literary search

**Fig. 1:**
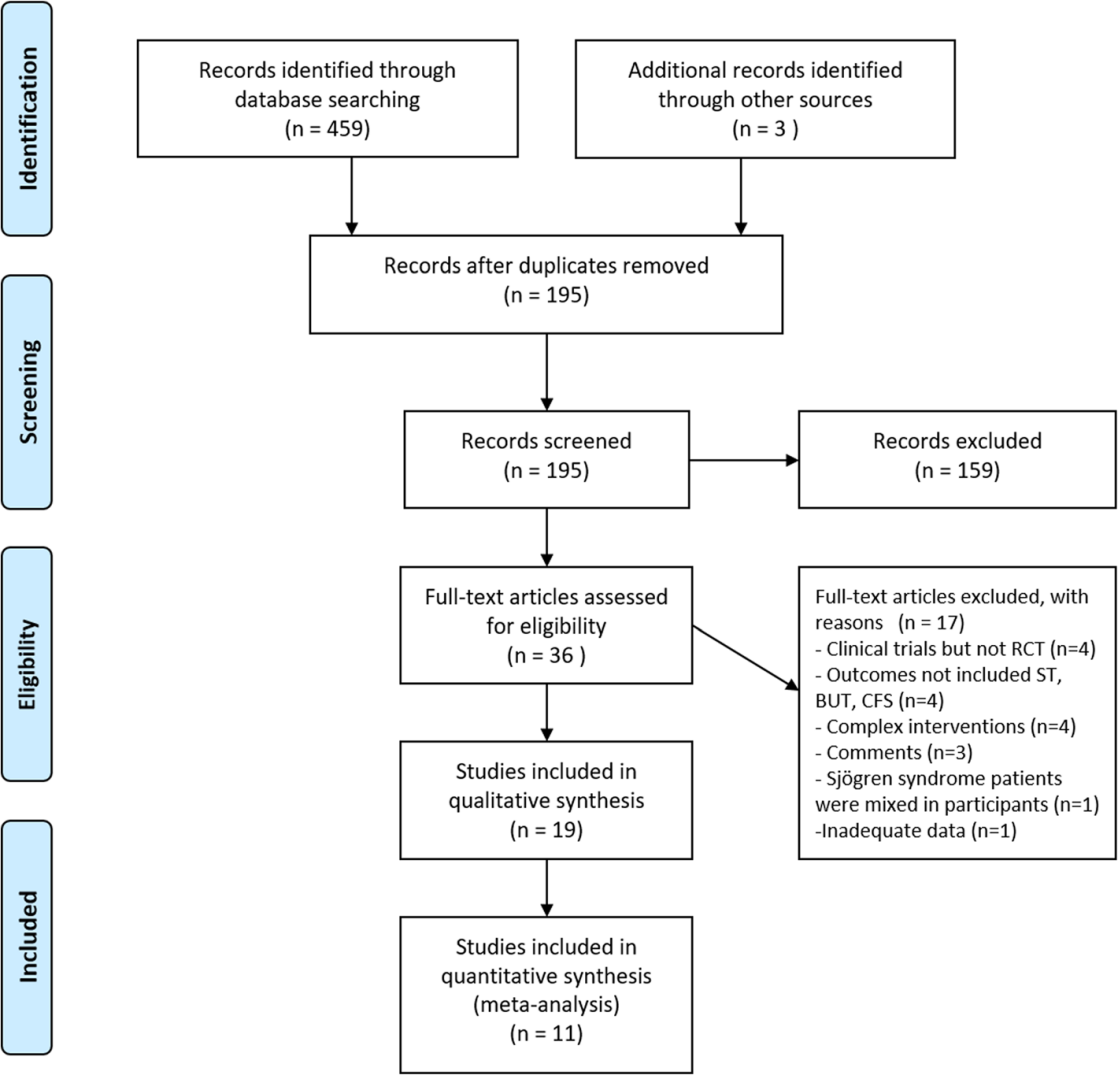
Flow diagram of the systemic process for report identification

We identified 462 articles of potential relevance. Screening the titles and abstracts yielded 36 studies. After reviewing the full texts, we selected 19 studies. Studies were excluded for the following reasons: (1) not an RCT (*n* = 4); (2) outcomes did not include ST, BUT, or CFS (n = 4); (3) acupuncture was part of a complex intervention (n = 4); (4) comments (*n* = 3); (5) inadequate data (*n* = 1); (6) Sjögren syndrome patients were included as participants (n = 1). The procedure is summarized in Fig. 1.

### Study description

We included 19 studies and 1126 subjects in this review. Fourteen studies [12–25] compared manual acupuncture with AT. One study [26] used sham acupuncture as a control group. Four studies [27–30] used an applied form of acupuncture (two studies used electroacupuncture, and two used pyonex). Fifteen of the studies [13, 14, 16–19, 21–25, 27–30] were conducted in China. The study descriptions are given in Tables 1 and 2.

**Table 1:** Characteristics of included studies (manual acupuncture vs. artificial tears)

First author	Year	Site	Sample size^a^	Age	Gender (M/F)	Regime (acupoints)	Duration (min)	Frequency (per week)	Total sessions	Outcomes
Nepp [12]	1998	Austria	52 (30/22)	N/A	N/A	GB1, BL2, ST5, EX-HN2, LI4, SI3, LI3, KI6, TE5	30	1	10	ST, BUT, drop frequency
He [13]	2004	China	32 (16/16)	52	12/20	Pattern identification was done by practitioner (ST2, LI20, LI11, LI4, SP6 OR ST2, SP10, SP9, SP6, ST36, KI6)	20–25	3–4 (every other day)	30	ST, BUT, CFS
Wang [14]	2005	China	45((A)15, (B)15/15)	51.7	17/28	(A) Pattern identification was done by practitioner (LI11, LI4 SP6, KI3, ST2, LI20 or ST2, SP10, SP9, ST36, SP6, ST40) (B) BL2, TE23, GB14, ST1	20–25	3–4 (every other day)	20	ST, BUT, CFS, RR
Tseng [15]	2006	Taiwan	26 (17/9)	48.9	12/14	Ex-HN5, TE23, GB14, ST2, SP6	20	2	16	ST, BUT, Number of application of artificial tears
Zhang [16]	2009	China	60 (30/30)	44	23/37	ST1, LR3, KI3	20	N/A	N/A	ST, BUT, total symptoms
Gong [17]	2010	China	42 (20/22)	44.8	11/33	BL1, BL2, GB14, SJ23, Ex-HN5, ST2, LI4, LR3, GB37, SP6, GB20	20	3 (every other day)	10	ST, BUT, RBS recording, total score
Gao [18]	2010	China	56 (28/28)	48.9	3/53	BL1, BL2, TE23, GB1,Ex-HN5	30	6	24	ST, BUT, total score
Shi [19]	2012	China	68 (33/35)	49.5	30/38	Ex-HN1, BL1, ST1, Ex-HN5, TE23, LI4, ST36	25	3	9	ST, BUT, tear lactoferrin concentration
Kim [20]	2012	Korea	150 (75/75)	42	41/109	BL2, GB14, TE23, Ex-HN5, ST1, GB20, LI4 LI11, GV23	20	3	12	OSDI, VAS, BUT, ST, MYMOP-2
Nan [21]	2014	China	60 (30/30)	48.1	25/35	Eye acupuncture (liver/gallbladder area, kidney area, spleen stomach area, upper jiao area)	15–20	7	20	ST, BUT, total score
Zhang [22]	2015	China	80 (40/40)	53	28/52	Hair needle therapy (superior and inferior lacrimal puncta)	10	3–4 (every other day)	7	ST, BUT, CFS, total score
NI [23]	2016	China	93((A)30, (B)32/31)	33.3	36/57	(A) BL1, Ex-HN7, SP6, KI3, GV26 (B) BL1, Ex-HN7, SP6, KI3	20	3	9	ST BUT, subjective symptom score
Chao [24]	2016	China	53((A)18, (B)19/16)	48.9	15/38	(A), (B) GB20, Ex-HN5, BL2, ST2, LR3, KI3, SP6, SP6, ST36, ST37 (A) applied a qi-absorption needling technique to the GB20	30	7	28	ST, BUT, VAS, CFS total score
Liu [25]	2017	China	28 (14/14)	60.7	0/28	BL2, BL3, TE23, Ex-HN5, ST2, LI4, GB20, GV20, ST1	30	3	24	ST, BUT, OSDI, questionnaire, protein analysis

*M/F* male/female, *ST* Schirmer’s test, *BUT* break-up time, *CFS* corneal fluorescein staining, *RBS* Rose-Bengal staining, *RR* response rate, *OSDI* ocular surface disease index, a questionnaire

^a^Total sample size (number who received manual acupuncture/number who received artificial tears)

**Table 2:** Characteristics of included studies (other kinds of interventions)

First author	Year	Site	Sample size^a^	Age	Gender (M/F)	Regime (intervention/control group)	Regime (frequency & duration)	Outcomes
Shin [26]	2010	Korea	42 (21/21)	41.6	11/31	Intervention: manual acupuncture GV23, BL2, BL14, TE23, Ex-HN5, ST-1, GB20, SP3, LU9, LU10, HT8 Control: non-acupoints around same site	Needles were retained for 20 min, 3 times a week for a total of 9 times. It takes 3 weeks to complete the treatment.	BUT, SIT, VAS, OSDI
Liu [27]	2012	China	39 (20/19)	32	21/19	Intervention: electro-acupuncture with BL1, Ex-HN5, BL2, TE23, GB1, GB20, KI3, SP6, LR3 Control: Artificial tears	Everyday treatment group undergoes electroacupuncture for 30 min. For a total of 20 sessions.	SIT, BUT
Guo [28]	2013	China	47 (23/24)	52	7/40	Intervention: electro-acupuncture with shang-jingming (Ex), xia-jingming (Ex), GB1, BL2, GB20, LI4, SP6, KI3, LR3 Control: manual acupuncture with the same acupoints	Needles were retained for 20 min, 3 times a week for a total of 12 times. It takes 4 weeks to complete the treatment.	BUT, SIT, VAS
Gao [29]	2016	China	88 (44/44)	41.8	33/55	A: pyonex combined with acupuncture, SP6, ST36, PC6, LR3 are the main points, and pattern identification was done (BL13, LI4 OR BL20, ST40 OR BL18, BL23) B: manual acupuncture with GV20, ST1, BL2, GB20, Ex-HN5, TE23	A: pyonex was embedded for 3 days. The follwing day, it was embedded again. This process was repeated four times and followed by two days free from embedment. After that, another four courses of embedment were conducted. B: needles were retained for 30 min every day for 12 days. Two days rest were followed by another 12 days of treatment.	SIT BUT, total score
Wu [30]	2016	China	40 (20/20)	44.2	10/30	Intervention: BL2, ST2, Ex-HN5 Control: artificial tears	Treatment group undergoes embedding therapy (retained for 24 h) on alternate days for 7 sessions.	BUT, SIT, OSDI

*M/F* male/female, *ST* Schirmer’s test, *BUT* break-up time, *OSDI* ocular surface disease index, a questionnaire

^a^Total sample size (number in intervention group/number in control group)

### Risk of bias assessment

**Fig. 2:**
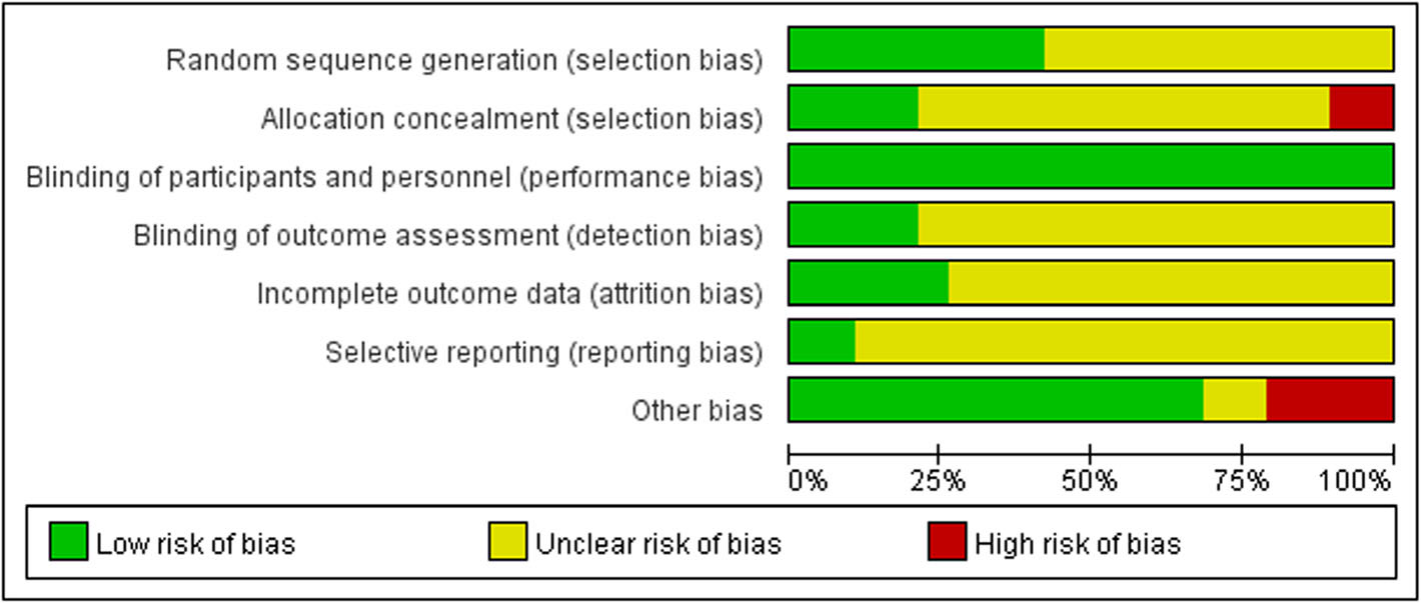
Risk of bias summary in included studies

In 8 studies [14, 16, 19–21, 23, 26, 28], the investigators described a method of random sequence generation (random number table, coin tossing, envelope shuffling, and using a computerized random-number generator). Only 4 studies [16, 20, 26, 28] conducted allocation concealment, and 4 studies [15, 19, 20, 28] used assessor blinding. Drop rates and reasons were reported in 6 studies [19, 20, 23, 26, 28]. Two studies used a study protocol [20, 26]. In 4 studies, we suspected bias: two studies [16, 17] had significant differences between the treatment and control groups at baseline without revision or explanation; in the other two studies, the duration differed between the treatment and control groups [13, 14]. Figure 2 summarizes the risk of bias assessment. Furthermore, there was no evidence of significant publication bias by inspection of the funnel plots.

### Meta-analysis between manual acupuncture and artificial tears

In this meta-analysis, we used the results of 11 of 14 studies that recorded both BUT and ST. We excluded Nepp [10] and Gong [15] because the former showed only graph and the later showed change value. We also excluded Zhang [22] through our sensitivity analysis because it presented a heterogeneous result that did not seem to be due to acupuncture. We did not consider CFS results in this meta-analysis because two studies used a dichotomous scale [13, 14], three used a continuous scale [18, 22, 23], and they used different methods of measuring.

**Fig. 3:**
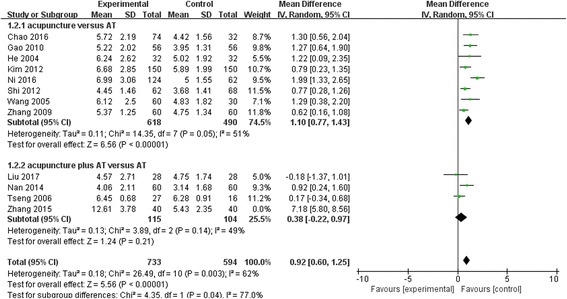
Break up time (BUT) comparison between acupuncture and artificial tears (AT): Random effect model

In the BUT results, a significant difference was shown between groups (WMD, 0.92; 95% CI, 0.60 to 1.25; *p* < 0.00001), but the heterogeneity was high (I^2^ = 62%; *P* = 0.003). The studies that used an acupuncture plus AT intervention did not show a mean difference (*p* = 0.21), but the studies that used only acupuncture did show a significant difference (p < 0.00001), including a subgroup difference(*p* = 0.04) (Fig. 3).

**Fig. 4:**
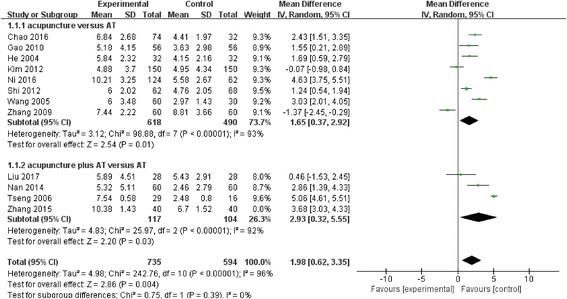
Schirmer’s test (ST) comparison between acupuncture and artificial tears (AT): Random effect model

The ST results also showed a significant difference between manual acupuncture and AT (WMD, 1.98; 95% CI, 0.62 to 3.35; *p* = 0.004), though again, the heterogeneity of the effect was high (I^2^ = 96%; *P* < 0.00001). Each of the two subgroups showed significant differences within the groups, but we did not find differences between the subgroups. (WMD, 1.65; 95% CI, 0.37 to 2.92 versus WMD, 2.93; 95% CI, 0.32 to 5.55; *p* = 0.39) (Fig. 4).

**Fig. 5:**
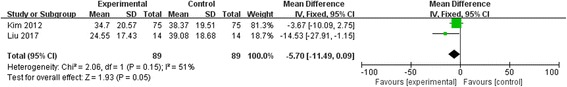
Ocular surface disease index (OSDI) comparison between acupuncture and artificial tears (AT): Fixed model

Two studies [20, 25] evaluated OSDI scores, and they showed no significant differences between groups (WMD, − 5.70; 95% CI, − 11.49 to 0.09; *p* = 0.05, Fig. 5).

### Subgroup analysis

In treatment duration and frequency, long-term (more than 1 month) and less frequent (less than 3 times a week) treatments were more effective than short-term or intensive treatments, but the difference was not significant. (Table 3).

**Table 3:** Results of subgroup analysis by treatment duration, frequency, and specific acupoints

Variables		Number of studies	Number of eyes		SMD	95%CI	Heterogeneity (I^2^)	MD (95% CI)
			Acupuncture	AT				
Duration	Short term	7	586	488	0.54	0.30–0.78	86%	−0.41 (−1.10, 0.28)
	Long term	4	148	106	0.95	0.30–1.59	87%	
Frequency	Less frequent	4	250	208	0.49	0.19–0.79	79%	−0.22 (−0.67, 0.22)
	Intensive	7	484	386	0.71	0.37–1.05	90%	
BL1	Included	4	270	214	0.60	0.29–0.91	81%	−0.04 (−0.49, 0.41)
	Not included	7	464	380	0.64	0.32–.0.96	89%	
BL2^a^	Included	4	264	264	0.35	0.11–0.60	71%	−0.46 (− 0.86, − 0.06)
	Not included	8	470	360	0.81	0.49–1.12	89%	
ST1^a^	Included	5	330	336	0.28	0.01–0.52	77%	−0.63 (−1.02, − 0.24)
	Not included	7	404	288	0.91	0.60–1.22	85%	
ST2	Included	4	148	106	0.95	0.30–1.59	91%	0.41 (−0.28, 1.10)
	Not included	7	586	488	0.54	0.30–0.78	86%	
TE23^a^	Included	6	354	348	0.59	0.25–0.96	88%	−0.10 (− 0.55, 0.35)
	Not included	6	380	276	0.69	0.40–0.99	87%	
Ex-HN5	Included	6	398	350	0.60	0.27–0.93	88%	−0.03 (−0.56, 0.50)
	Not included	5	336	244	0.63	0.30–0.96	86%	

^a^Wang et al. [15] study consists of two different acupuncture group. One group included BL2, ST1, TE23 and the other did not. For the reason, number of studies and number of eyes of AT group was double-counted

In acupoint differences, regimes including the BL2 or ST1 acupoints showed significantly weaker effects than regimes that did not include them (BL2; SMD, 0.35 vs 0.81; *p* = 0.03, ST1, SMD, 0.28 vs 0.91; *p* = 0.002). There were no significantly different values for other acupoints (BL1, TE23, ST2, Ex-HN5, Table 3).

### Other interventions

One study compared verum acupuncture with sham acupuncture [26]. In this study, the sham acupuncture was applied to non-acupoints (peri-acupoints). There were no significantly different outcomes between the verum and sham acupuncture groups. Two studies used electroacupuncture: Liu et al. compared electroacupuncture with AT and found significant ST and BUT effects in the experimental group [27]; Guo et al. compared electroacupuncture at BL1 with manual acupuncture and found minor effects in eye symptoms and ST in the intervention group [28]. Two studies investigated pyonex: one study compared pyonex with manual acupuncture [29], and the other compared it with AT and found limited improvements compared with the control group [30] (Table 2).

## Discussion

Some previous systematic reviews have considered the effects of acupuncture in DES [3–5]. In this study, we focused on subgroup analysis to solidify the results and suggest an effective method of acupuncture treatment. We excluded one study [22] for heterogeneity because it showed a unique change and used an unusual intervention: the practitioners treated the superior/inferior lacrimal punctum and proceeded horizontally 5-15 mm through the lacrimal canaliculi, similar to lacrimal probing. This effect focused on penetration, so it was difficult to regard its effects as resulting purely from the acupuncture.

As in former studies, heterogeneity in the effects of manual acupuncture was high; we conducted subgroup analysis for method of intervention, treatment duration, frequency, and acupoints. However, accounting for those factors did not lower the heterogeneity. A high risk of bias can cause heterogeneity. Information about the randomization method, number of and reason for drop outs, statistical analysis methods, and blinding outcome analysis is essential to decrease the risk of bias in a clinical study. However, several studies did not include this data.

Acupuncture combined with AT treatment had a weaker effect on BUT score but a stronger effect on ST score and a better overall effect than acupuncture treatment alone. ST evaluates tear production, and BUT evaluates tear film stabilization. Acupuncture plus AT thus showed a synergetic effect in tear production but not in tear stabilization. Manual acupuncture affected the protein composition in tears [19, 25], but combining with AT, there was no synergetic effect, which clinicians should consider when planning treatments for individual patients.

With regard to duration and frequency, a long period of treatment (more than 1 month) is preferable to a short period (less than 1 month), but frequent treatment does not guarantee better effects. Three times a week showed better effectiveness than 5–6 times a week. Recovery time might be needed to produce optimal effects. Harris et al. said that the effects of acupuncture are dose dependent [31]. Experts recommend at least twice a week to obtain a proper effect [32]. Therefore, 2–3 times a week could be an optimal frequency to maximize the effects.

Most studies we selected included periorbital acupoints. Thus, the effects of acupuncture in DES are mostly local. According to Shin et al. [26], the effect of acupuncture on DES occurs by dilating blood vessels and increasing the supply of neuropeptides (e.g., Calcitonin gene related peptide). However, our analysis implied positional specificity. The studies that included points BL2 and ST1 were less effective than those that did not include those points. This could be related to the analgesic effect of acupuncture. ST1 is located between the eyeball and the infraorbital ridge, directly below the pupil, and BL2 is located at the medial end of the eyebrow. They are thus near the supraorbital nerve block and infraorbital nerve block sites. Those two nerves innervate the conjunctiva, and decreased conjunctiva sensitivity decreases tear production. Therefore, clinicians should select acupoints according to patient symptoms.

Some of the included studies also considered electroacupuncture and pyonex. Electroacupuncture is commonly considered to be a stronger stimulus than acupuncture. Pyonex is a kind of patch acupuncture known for its long retention. However, only a few studies used those interventions, and they had many limitations in design. Therefore, we did not evaluate the effects of electroacupuncture and pyonex in DES. Well-designed studies are needed to evaluate their effects.

Additionally, Female sex is a risk factor in DES [33]. In our study, there were also female subjects than male subjects. Sex hormone (e.g., androgen) can be a factor in explaining this result. Androgen have a positive effect as anti-inflammatory effect on DES [34, 35]. Acupuncture may modulate sex hormone [36]. However, there was no study focusing on this point. Further study concerning about the hormone is needed to clarify the mechanism of acupuncture.

Our study has several limitations. We conducted indirect comparisons and used the Bucher method to avoid some biases. Indirect comparisons require more caution in interpretation than direct comparisons [37]. Moreover, they require homogeneity for validity. However, the analyzed studies were so heterogeneous in their subgroups that bias was almost inevitable. Nonetheless, even though an indirect analysis is less reliable than a direct comparison, it can allow doctors to make treatment plans for patients and defines a method to improve treatment effects and reproducibility.

Furthermore, some of the reviewed studies have significant weak points. Some studies that we analyzed did not report information necessary to discern the risk of bias. Comparing acupuncture with AT is an open label trial. Therefore, strict randomization, allocation concealment, and blinding of outcome assessments are needed to avoid bias. Furthermore, only two studies conducted [19, 20] follow-up evaluations. Although manual acupuncture was more effective than AT, AT is more convenient to use. Subjects who want to receive acupuncture treatment must go to a clinic and spend time there, which is not necessary for AT treatment. Therefore, acupuncture must have strengths that outweigh the convenience of AT. One study suggested that acupuncture effects last longer than AT [20]. The disadvantage of typical AT is its short persistence. Further studies should include follow-up evaluations to strengthen this conclusion.

## Conclusions

Acupuncture is more effective than AT in DES. Treatment duration of more than 1 month and treatment frequency less than 3 times a week could be more effective than shorter or more frequent treatments. Use of the BL2 and ST1 acupoints can reduce the overall effectiveness of acupuncture for DES. Other acupuncture treatments (electroacupuncture, pyonex) could be applicable, but there are not yet enough studies to evaluate their effects.

## Abbreviations

AT: Artificial tears
BUT: Break-up time
CFS: Corneal fluorescein staining
DES: Dry eye syndrome
RCT: Randomized controlled trials
ST: Schirmer’s test
